# Efficacy and safety of electroacupuncture for post-stroke depression: a systematic review and meta-analysis

**DOI:** 10.3389/fneur.2025.1732787

**Published:** 2026-01-12

**Authors:** Yue Liang, Yujie Gu, Hui Han, Huichao Yin, Junjun Gao, Libo Hou, Wenwen Ning, Zuncheng Zheng

**Affiliations:** 1 Rehabilitation Centre, Taian Central Hospital, Taian, China; 2 Rehabilitation Centre, Qingdao University Affiliated Taian Central Hospital, Taian, China; 3 Rehabilitation Centre, Taishan Medical and Care Centre, Taian, China

**Keywords:** depression, electroacupuncture, meta-analysis, stroke, systematic review

## Abstract

**Background:**

Depression is a common comorbid condition in stroke patients, significantly impairing their quality of life. Electroacupuncture (EA) has shown promising effects in treating post-stroke depression (PSD), but integrated evidence remains scarce.

**Objective:**

To investigate the efficacy of EA in improving PSD and evaluate its clinical effectiveness and safety through systematic review and meta-analysis.

**Methods:**

We searched eight electronic databases (PubMed, Cochrane Library, Embase, Web of Science, CNKI, VIP Data Platform, Wanfang Data Knowledge Service Platform, and China Biomedical Literature Service System) and manually reviewed reference lists of relevant literature and clinical trial registries for randomized controlled trials (RCTs) of EA for PSD. Eligible studies were screened based on inclusion and exclusion criteria, and relevant data were extracted. Meta-analysis was performed using RevMan 5.4 software.

**Results:**

A total of 22 studies involving 1,640 patients were included. Meta-analysis results showed that EA improved HAMD (MD = −1.71, 95% CI: −2.79 to −0.63, *I*^2^ = 89%, *p* = 0.002), efficacy rate (OR = 1.94, 95% CI: 1.43 to 2.64, *I*^2^ = 0%, *p* < 0.0001), and safety (OR = 0.2, 95% CI: 0.12–0.34, *I*^2^ = 45%, *p* < 0.00001). However, it did not show superiority over the control group in terms of the Barthel Index (BI) (MD = 4.01, 95% CI: −0.26 to 8.27, *I*^2^ = 70%, *p* = 0.07) and SDS (MD = −3.28, 95%CI: −6.78 to 0.21, *I*^2^ = 97%, p = 0.07). The GRADE assessment indicates that the evidence level for HAMD is very low, while that for BI is low.

**Conclusion:**

EA demonstrated superiority over conventional drug therapy in improving HAMD scores, efficacy rates, and safety. However, no significant difference was observed between EA and the control group in improving BI and SDS scores. The pooled results for HAMD scores and SDS showed high heterogeneity, and the study itself exhibited considerable heterogeneity. Furthermore, all included studies were conducted in China, which may introduce regional bias. Future rigorous clinical research is needed to provide high-quality evidence.

## 1 Introduction

Stroke is characterized by high incidence, high disability rates, and high mortality, significantly impacting global health (1). Post-stroke depression (PSD), as a common and severe psychiatric complication, adversely affects patients’ recovery and quality of life (2). Data indicates that 25.4% of patients develop PSD within 2 years of stroke onset, yet only 7.8% recognize their depressive symptoms (3). These individuals often lack timely or standardized treatment. Despite physical functional improvements, psychological symptoms may worsen, hindering overall recovery.

PSD onset is associated with factors like age, gender, education level, and stroke type. It commonly manifests as persistent low mood, accompanied by cognitive impairment, apathy, anhedonia, fatigue, self-blame, self-harm behaviors, and even suicidal ideation (4). PSD exhibits complex interactions with other complications such as anxiety, neurological deficits, and obstructive sleep apnea (5), potentially leading to severe functional impairment, reduced quality of life, recurrent stroke, and mortality (6, 7). PSD may involve diverse etiological mechanisms, encompassing biological and psychological hypotheses (8). Biological hypotheses include four mechanisms: lesion localization, neurotransmitter, inflammatory cytokine, and genetic polymorphism. The specific location of lesions plays a crucial role in PSD pathogenesis; reduced serotonin and norepinephrine in the brain are associated with PSD; increased cytokines post-stroke may lead to depression; and the short variant genotype of the serotonin transporter gene linkage promoter region shows a significant association with major depressive disorder after stroke (9). Psychological hypotheses suggest that social and psychological stressors associated with stroke may be primary contributors to depression. Currently, no definitive evidence supports or refutes purely biological or purely psychosocial mechanisms. It appears to be a psychosocial, multifactorial mental disorder.

Current PSD treatment primarily relies on pharmacotherapy, supplemented by psychotherapy and techniques like transcranial magnetic stimulation (10). However, pharmacological interventions exhibit slow onset (typically 4–8 weeks) and are prone to drug tolerance and adverse reactions (11, 12). This poses challenges for PSD patients’ recovery. EA is a technique that builds upon traditional acupuncture methods. After obtaining qi sensation at the acupoint, a microcurrent wave (sensing) that responds to the body’s bioelectricity is applied through the needle to treat diseases. It is currently widely used in the treatment of PSD and has achieved certain clinical effects. Although clinical evidence demonstrates the good efficacy of EA in treating PSD, the current evidence has not been effectively integrated and has not formed persuasive evidence-based medical evidence.

Therefore, this study conducts a systematic review and meta-analysis of clinical research on EA for PSD to integrate existing evidence, provide reliable guidance for clinical practice, clarify current research limitations, and offer reference for future studies.

## 2 Materials and methods

This study was conducted according to the PRISMA statement and was registered in PROSPERO prior to commencement (Registration Number: CRD420251171897).

### 2.1 Data sources and search strategy

This study searched eight databases including PubMed, Cochrane Library, Embase, Web of Science, China Knowledge Infrastructure (CNKI), China Science Journal Database (VIP), Wanfang Database, and China Biomedical Literature Service System (CBM). Publications were limited to English and Chinese languages, with the search cutoff date set at July 21, 2025. Mesh terms were combined with free-text keywords for retrieval, with primary search terms including “Electroacupuncture,” “Stroke,” and “Depression.” The specific search strategy is detailed in Table 1 (using PubMed as an example). Search strategies for other databases and the PRISMA 2020 checklist are provided in the Supplementary material.

**Table 1:** Search strategy (PubMed as an example).

Rank	Search term	Result
#1	Electroacupuncture [MeSH]	5,658
#2	Electroacupuncture OR electro-acupuncture OR electric acupuncture OR Electroneedle OR “Electroacupuncture Treatment” OR “Treatment, Electroacupuncture”	11,276
#3	#1 OR #2	11,276
#4	Stroke [MeSH]	192,769
#5	Ischemic stroke [MeSH]	18,031
#6	Hemorrhagic stroke [MeSH]	844
#7	Brain infarction [MeSH]	45,224
#8	Cerebral hemorrhage [MeSH]	39,969
#9	Cerebrovascular accident OR Cerebrovascular Accidents OR CVA OR Cerebrovascular Apoplexy OR Apoplexy, Cerebrovascular OR Vascular Accident, Brain OR Brain Vascular Accident* OR Vascular Accidents, Brain OR Cerebrovascular Stroke* OR Stroke, Cerebrovascular OR Strokes, Cerebrovascular OR Apoplexy OR Cerebral Stroke* OR Stroke, Cerebral OR Strokes, Cerebral OR Stroke, Acute OR Acute Stroke* OR Strokes, Acute OR Cerebrovascular Accident, Acute OR Acute Cerebrovascular Accident* OR Cerebrovascular Accidents, Acute	505,119
#10	#4 OR #5 OR #6 OR #7 OR #8 OR #9	531,500
#11	Depression [MeSH]	282,884
#12	Depressive disorder [MeSH]	129,269
#13	Depressive Symptoms OR Depressive Symptom OR Symptom, Depressive OR Emotional Depression OR Depression, Emotional OR Depressive Disorders OR Disorder*, Depressive OR Neurosis, Depressive OR Depressive Neuroses OR Depressive Neurosis OR Neuroses, Depressive OR Depression*, Endogenous OR Endogenous Depression* OR Depressive Syndrome* OR Syndrome*, Depressive OR Depression*, Neurotic OR Neurotic Depression* OR Melancholia* OR Unipolar Depression OR Depression*, Unipolar OR Unipolar Depressions	637,288
#14	#11 OR #12 OR #13	637,288
#15	#3 AND #10 AND #14	50

### 2.2 Inclusion and exclusion criteria

Inclusion criteria were established using the PICOS (Population, Intervention, Comparison, Outcome, Study Design) framework. Specific criteria were: (1) P: Participants with a confirmed diagnosis of PSD; (2) I: Treatment group receiving EA therapy; (3) C: Control group receiving conventional medication; (4) O: Using the HAMD as the primary indicator; (5) S: Study design being randomized controlled trials.

Exclusion criteria: (1) Studies with unclear diagnoses or comorbidities affecting outcome assessment; (2) Studies involving concurrent interventions; (3) Studies with non-representative outcome measures; (4) Not randomized controlled trials; (5) Studies lacking full-text access or incomplete data.

### 2.3 Literature management and data extraction

Two researchers independently screened and extracted data based on inclusion and exclusion criteria. Disagreements were resolved through consultation with a third researcher. The screening process involved: (1) Removing duplicate records using EndNote X9.3 literature management software. (2) Reviewing titles and abstracts to exclude reviews, theses, conference papers, scientific achievements, and other non-relevant literature. (3) Full-text review to determine eligibility.

After literature screening, the following information was extracted: (1) Basic literature information: author details, publication year, etc.; (2) Basic subject information; (3) Intervention method, intervention frequency, and treatment duration; (4) Outcome measures.

### 2.4 Literature quality assessment

We used the Cochrane Risk of Bias tool (ROB 2.0) to assess the quality of included studies. This tool evaluates five domains: Bias in the randomization process, bias in deviation from the intended intervention, bias in missing outcome data, bias in outcome measurement, and bias in selective reporting of results. The bias in deviation from the intended intervention domain is further subdivided into two scenarios based on research objectives: evaluating the effectiveness of intervention assignment and evaluating the effectiveness of intervention adherence. Each domain contains multiple signal questions. When assessing the risk of bias in RCTs, researchers must make judgments and objectively answer these questions. Signal questions typically offer five response options: Yes (Y), Probably Yes (PY), Probably No (PN), No (N), and No Information (NI).

### 2.5 Statistical analysis

Following the recommendations of the Cochrane Collaboration and PRISMA guidelines, statistical analyses were conducted using Review Manager 5.4.1, reporting pooled risk ratios (RR) and mean differences (MD) with 95% confidence intervals (CI). Heterogeneity was quantified using the *I*^2^ statistic. Heterogeneity was defined as low, moderate, or high based on *I*^2^ values of 25, 50, and 75%, respectively. Publication bias for primary outcomes was visually assessed using funnel plots. The Egger regression test was applied to analyze publication bias.

To more accurately assess the robustness of the analysis results and the inclusion of bias in the studies, we shall employ Stata 18 to conduct sensitivity analyses and Egger’s test.

## 3 Results

### 3.1 Search results

**Figure 1:**
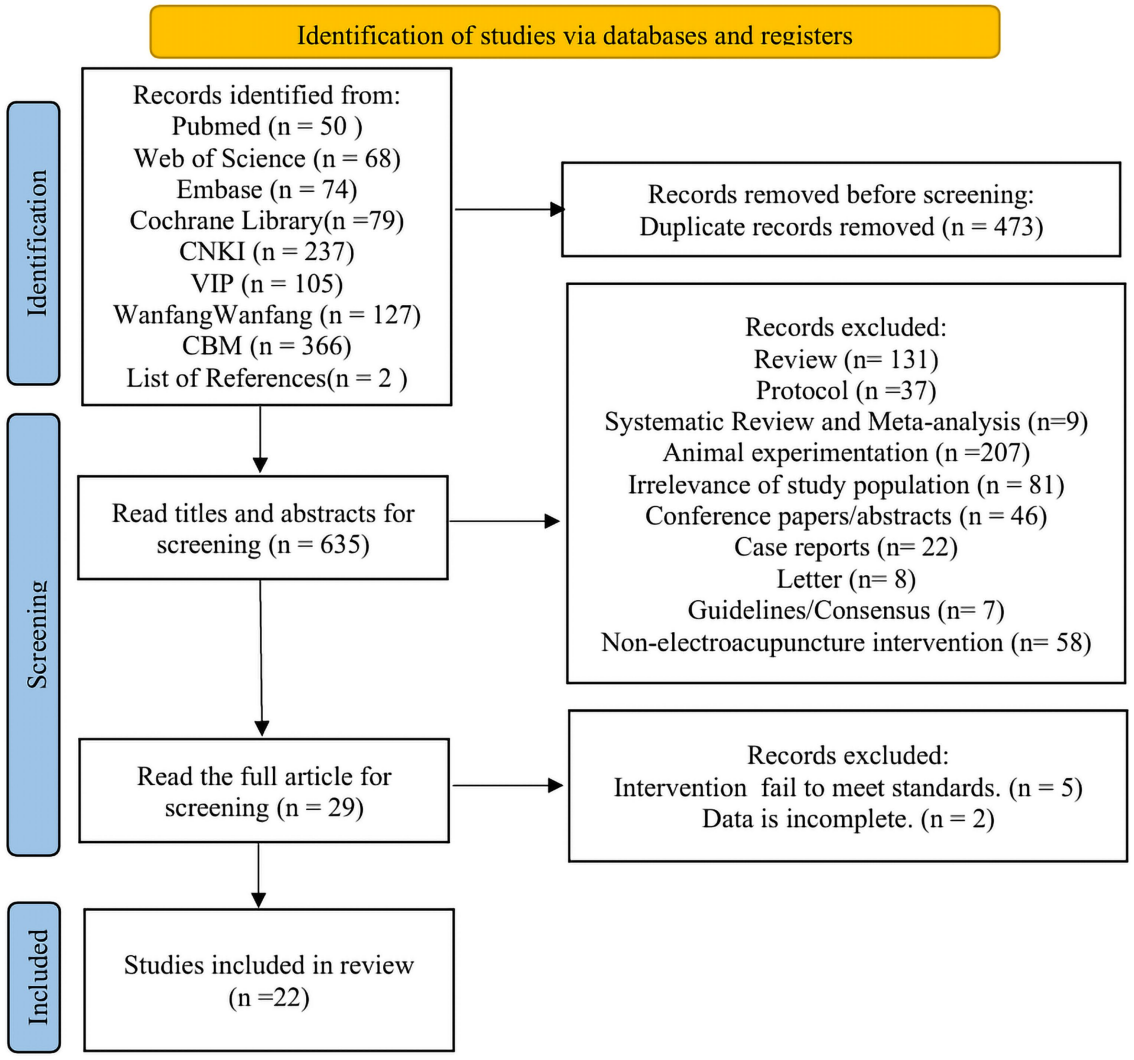
Literature screening process.

A total of 1,106 publications were retrieved from databases. After removing duplicates, 633 publications remained. Further screening based on title and abstract review yielded 27 publications. Full-text review of these 27 publications resulted in 22 eligible publications meeting inclusion criteria, all in Chinese. The specific screening process is illustrated in Figure 1.

### 3.2 Basic characteristics of included studies

A total of 20 studies were included (13–34). These comprised 22 individual research projects involving 1,761 patients. All studies were conducted in China across 12 provinces. All studies provided specific diagnostic criteria. The intervention group in each trial received EA. Among the control groups, 18 studies used fluoxetine as the control, two studies used sertraline tablets, one study used Flupentixol and Melitracen Tablets, and one study used sertraline hydrochloride. The intervention periods ranged from 4 weeks to 60 days across the 22 studies. Detailed information is presented in Table 2.

**Table 2:** Basic characteristics of included studies.

No.	Author	Year	Research location	Diagnostic criteria	Grouping method	Blind method	Sample size	Intervention methods	Intervention frequency	Course of treatment	Outcome
1	Bi et al.	2010	Shandong Province	Yes	Merely mentioned randomly	Double-blind	I:31 C:32	I: Electroacupuncture C: Fluoxetine	I: 5 t/w C: 20 mg/d	I:6 w C:6 w	HAMD; SDS
2	Chen	2012	Zhejiang Province	Yes	Merely mentioned randomly	Not mentioned	I:47 C:47	I: Electroacupuncture C: Fluoxetine	I: 3 t/w C: 20 mg/d	I:4 w C:4 w	HRDS; clinical efficacy rate; comparison of limb function
3	Chen et al.	2005	Jilin Province	Yes	Computerised random grouping	Not mentioned	I:30 C:30	I: Electroacupuncture C: Fluoxetine	I: 1 t/d C: 20 mg/d	I:4 w C:4 w	Clinical efficacy rate; SDS; SCAG
4	Chu et al.	2007	Jilin Province	Yes	Random number table method	Not mentioned	I:36 C:36	I: Electroacupuncture C: Fluoxetine	I: 5 t/w C: 20 mg/d	I:8 w C:8 w	HAMD; clinical efficacy rate; adverse events
5	Dai	2009	Hubei Province	Yes	Randomised allocation according to order of presentation	Not mentioned	I:30 C:30	I: Electroacupuncture C: Flupentixol and Melitracen Tablets	I: 6 t/w C: 21 mg/d	I:4 w C:4 w	HADM; clinical efficacy rate; onset time
6	Dong et al.	2017	Heilongjiang Province	Yes	Random number table method	Not mentioned	I:50 C:50	I: Electroacupuncture C: Fluoxetine	I: 1 t/d C: 20 mg/d increased to 80 mg/d	I:30 d C:28 d	HAMD; SDS; electroencephalogram changes; 5-HIAA; clinical efficacy rate
7	Dong et al.	2007	Heilongjiang Province	Yes	Random number table method	Not mentioned	I:38 C:34	I: Electroacupuncture C: Fluoxetine	I:1 t/d C: 20 mg/d	I:30 d C:28 d	HAMD; SDS; severity index; clinical efficacy rate
8	Hong et al.	2015	Zhejiang Province	Yes	Random number table method	Not mentioned	I:30 C:30	I: Electroacupuncture C: Escitalopram Oxalate Tablets	I: 6 t/w C: 20 mg/d	I:30 d C:30 d	HAMD; Barthel; clinical efficacy rate
9	Huang et al.	2014	Sichuan Province	Yes	Random number table method	Envelope method	I:30 C:30	I: Electroacupuncture C: Fluoxetine	I: 5 t/w C: 20 mg/d	I:6 w C:6 w	HAMD; Ronneberg Sleep Scale; Clinical efficacy rate; adverse events
10	Kang et al.	2014	Hebei Province	Yes	Merely mentioned randomly	Not mentioned	I:40 C:40	I: Electroacupuncture C: Fluoxetine	I: 1 t/d C: 20 mg/d	I:4 w C:4 w	NIHSS; ADL; HAMD
11	Li et al.	2015	Zhejiang Province	Yes	Computerised random grouping	Not mentioned	I:11 C:10	I: Electroacupuncture C: Fluoxetine	I: 1 t/d C: 20 mg/d	I:8 w C:8 w	HAMD; r CBF; clinical efficacy rate; ROI radioactive count ratio
12	Li et al.	2013	Heilongjiang Province	Yes	Randomised allocation according to order of presentation	Not mentioned	I:35 C:35	I: Electroacupuncture C: Fluoxetine	I: 1 t/d C: 20 mg/d	I:4 w C:4 w	HAMD; MESSS; clinical efficacy rate
13	Long et al.	2004	Hunan Province	Yes	Merely mentioned randomly	Single-blind	I:36 C:36	I: Electroacupuncture C: Fluoxetine	I: 6 t/w C: 10-40 mg/d	I:4 w C:4 w	HAMD; CNS; BI; FMA; adverse events
14	Nie et al.	2010	Guangdong Province	Yes	Random number table method	Not mentioned	I:30 C:30	I: Electroacupuncture C: Fluoxetine	I: 1 t/d C: 20 mg/d	I:60 d C:60 d	Clinical efficacy rate; HAMD; MESSS; Barthel
15	Peng et al.	2011	Guangdong Province	Yes	Merely mentioned randomly	Envelope Method	I:58 C:59	I: Electroacupuncture C: Fluoxetine	I: 6 t/w C: 20 mg/d	I:4 w C:4 w	HRSD; SDS; FIM; adverse events
16	Wang	2010	Zhejiang Province	Yes	Merely mentioned randomly	Not mentioned	I:40 C:40	I: Electroacupuncture C: Sertraline hydrochloride	I: 1 t/d C: 50 mg/d	I:30 d C:30 d	HAMD; affected side FMA
17	Wu et al.	2007	Gansu Province	Yes	Randomised allocation according to order of presentation	Not mentioned	I:32 C:31	I: Electroacupuncture C: Escitalopram Oxalate Tablets	I: 5 t/w C: 20 mg/d	I:6 w C:6 w	Clinical efficacy rate; HAMD;ADL; adverse events; TESS
18	Xu et al.	2015	Liaoning Province	Yes	Randomised allocation by treatment method	Not mentioned	I:40 C:40	I: Electroacupuncture C: Fluoxetine	I: 1 t/d C: 20 mg/d	I:6 w C:6 w	Clinical efficacy rate; HAMD
19	Zhang et al.	2013	Hubei Province	Yes	Merely mentioned randomly	Not mentioned	I:34 C:27	I: Electroacupuncture C: Fluoxetine	I: 6 t/w C: 20 mg/d	I:6 w C:6 w	HAMD; SDS
20	Zhou	2010	Henan Province	Yes	Computerised random grouping	Not mentioned	I:145 C:148	I: Electroacupuncture C: Fluoxetine	I: 1 t/d C: 20 mg/d	I:8 w C:8 w	HAMD; clinical efficacy rate; adverse events
21	Zhou	2007	Hunan Province	Yes	Merely mentioned randomly	Not mentioned	I:31 C:30	I: Electroacupuncture C: Fluoxetine	I: 1 t/d C: 20 mg/d	I:4 w C:4 w	HAMD; Adverse events; TESS; patient satisfaction rate
22	Zhu et al.	2012	Zhejiang Province	Yes	Merely mentioned randomly	Not mentioned	I:32 C:30	I: Electroacupuncture C: Fluoxetine	I: 5 t/w C: 20 mg/d	I:8 w C:8 w	Clinical efficacy rate; HAMD; adverse events

### 3.3 Quality assessment of included studies

**Figure 2:**
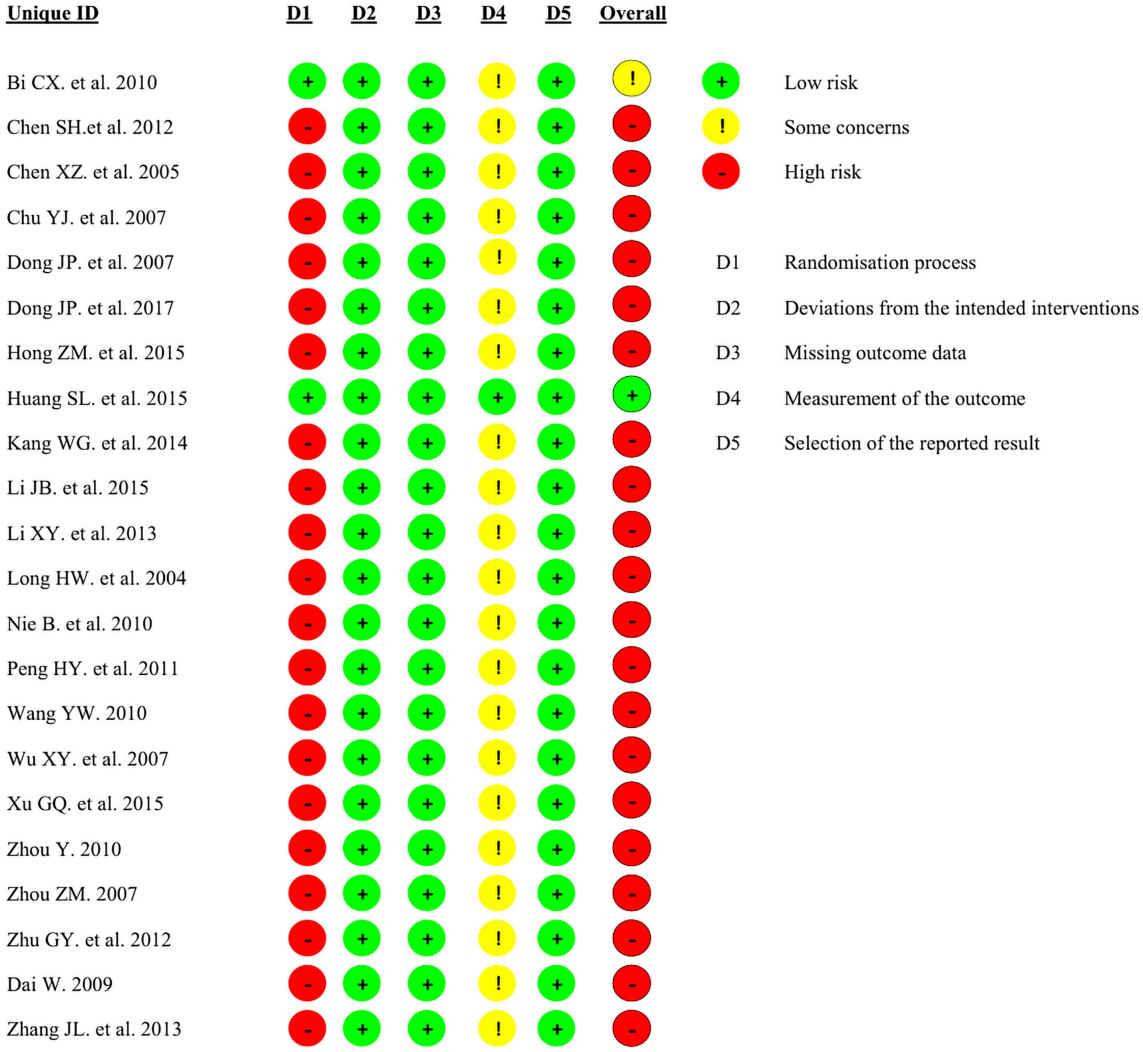
Risk of bias summary.

**Figure 3:**
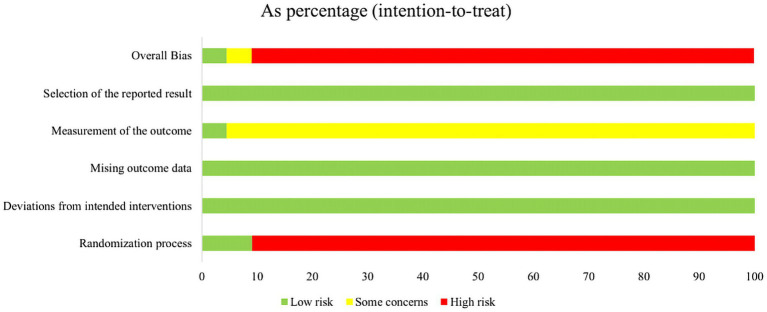
Risk of bias graph.

The Cochrane Risk of Bias tool (ROB 2.0) was used to assess the risk of bias in the included studies. Six studies used random number tables for allocation, three used computer software, 11 mentioned random allocation only, and two allocated participants based on order of arrival. One study employed an envelope method during randomization to ensure blinding. One study reported achieving double-blinding during the research process, one study reported achieving single-blinding, and the remaining studies mentioned blinding measures. This lack of consistent blinding protocols was the primary factor reducing study quality. Of the 22 studies, one was classified as “low risk,” one as “some concerns,” and 20 as “high risk.” Specific details are shown in Figures 2, 3.

### 3.4 Primary outcome measures

#### 3.4.1 Hamilton depression scale (HAMD)

**Figure 4:**
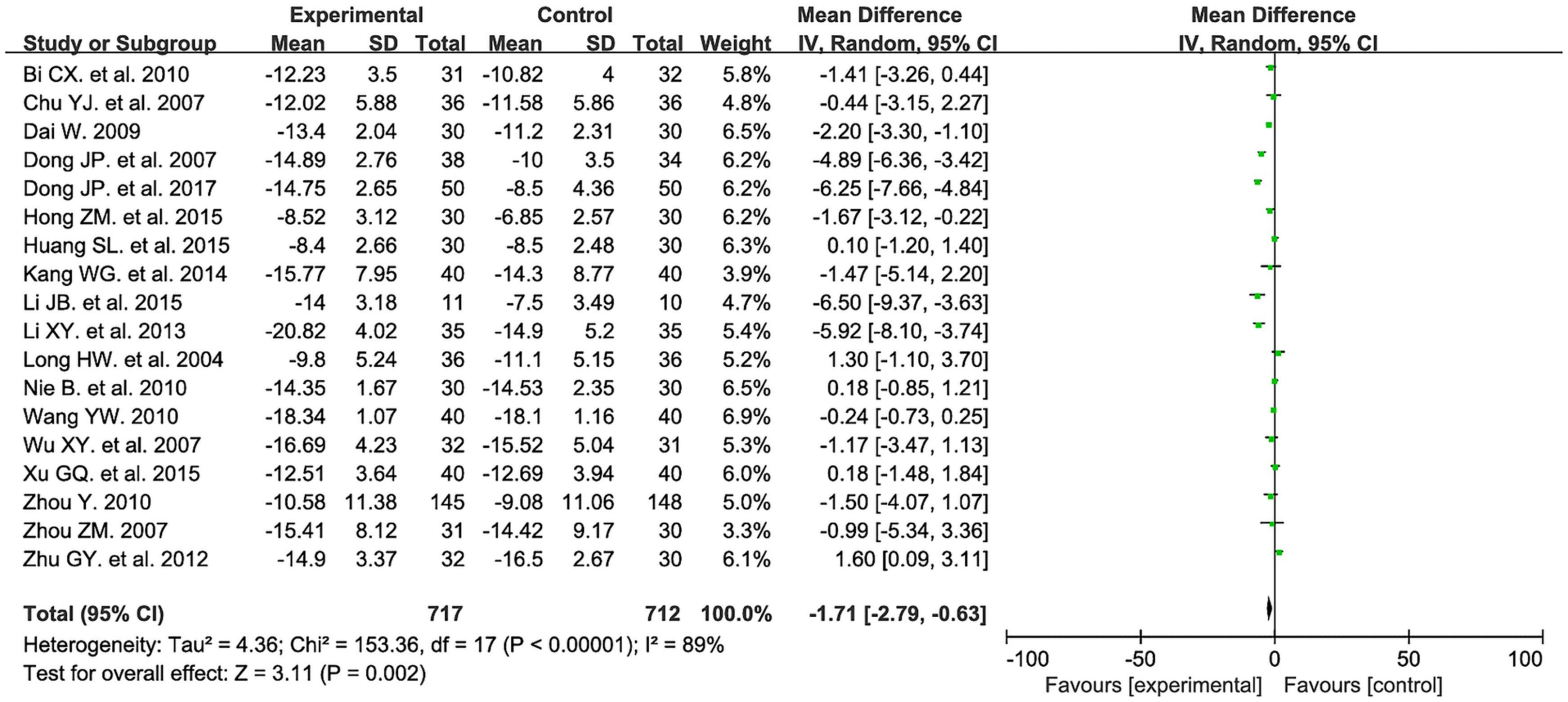
HAMD forest plot.

Eighteen studies evaluated HAMD scores before and after treatment, involving a total of 1,429 patients: 717 in the experimental group and 712 in the control group. Meta-analysis results indicated that the treatment group demonstrated greater improvement in HAMD scores compared to the control group (MD = −1.71, 95% CI: −2.79 to −0.63, *I^2^* = 89%, *p* = 0.002). See Figure 4 for details.

#### 3.4.2 Clinical efficacy rate

**Figure 5:**
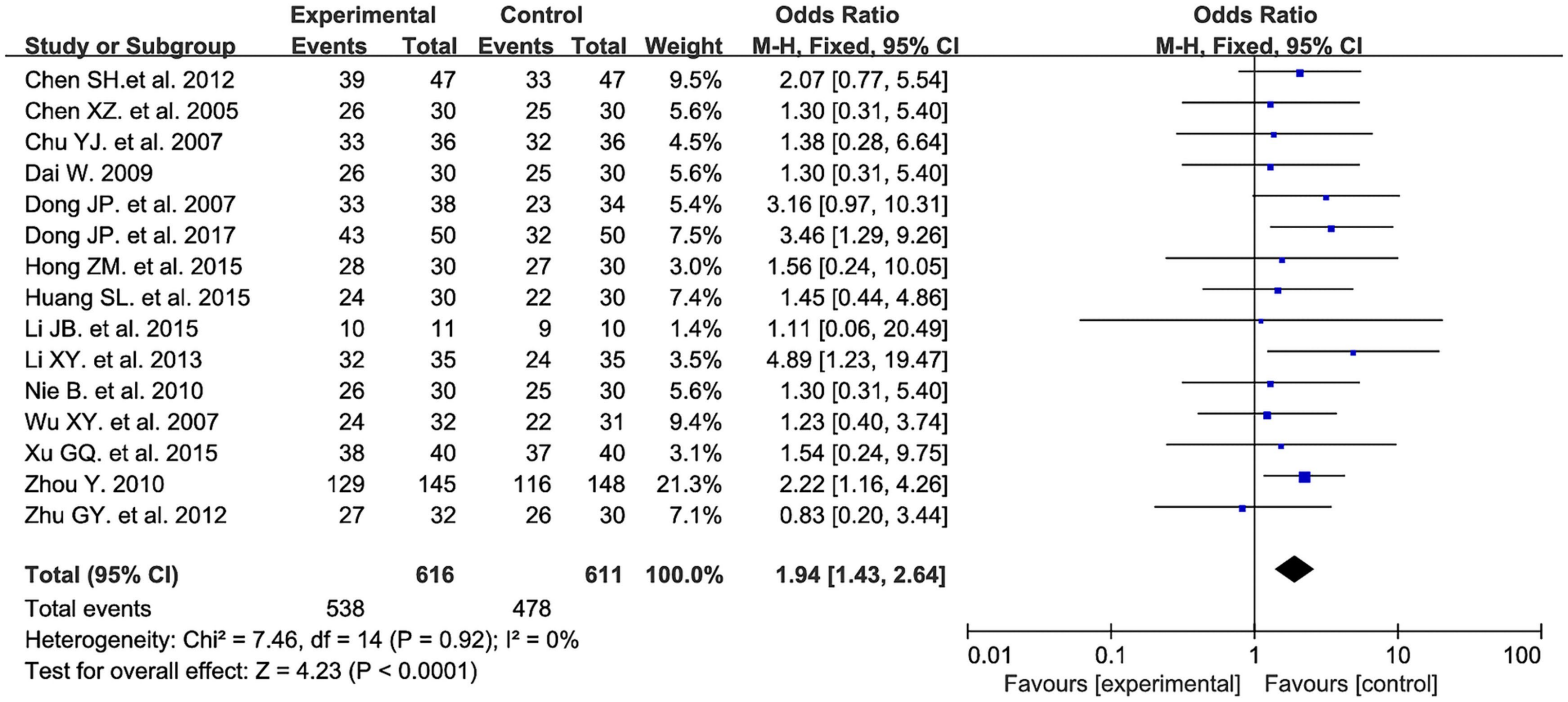
Clinical efficacy rate forest plot.

Fifteen studies evaluated the clinical efficacy rate between the two groups, involving a total of 1,227 patients: 616 in the experimental group and 611 in the control group. Meta-analysis results showed that the experimental group had a higher efficacy rate compared to the control group (OR = 1.94, 95% CI: 1.43 to 2.64, *I^2^* = 0%, *p* < 0.0001). See Figure 5 for details.

### 3.5 Secondary outcomes

#### 3.5.1 BI

**Figure 6:**
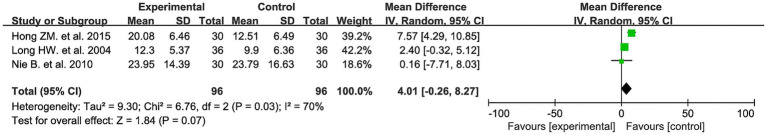
Barthel forest plot.

Three studies evaluated the BI before and after treatment, involving 192 patients (96 in the experimental group and 96 in the control group). The meta-analysis showed no significant difference between the two groups in improving the Barthel Index (MD = 4.01, 95% CI: −0.26 to 8.27, *I^2^* = 70%, *p* = 0.07). See Figure 6 for details.

#### 3.5.2 Self-rating depression scale (SDS)

**Figure 7:**
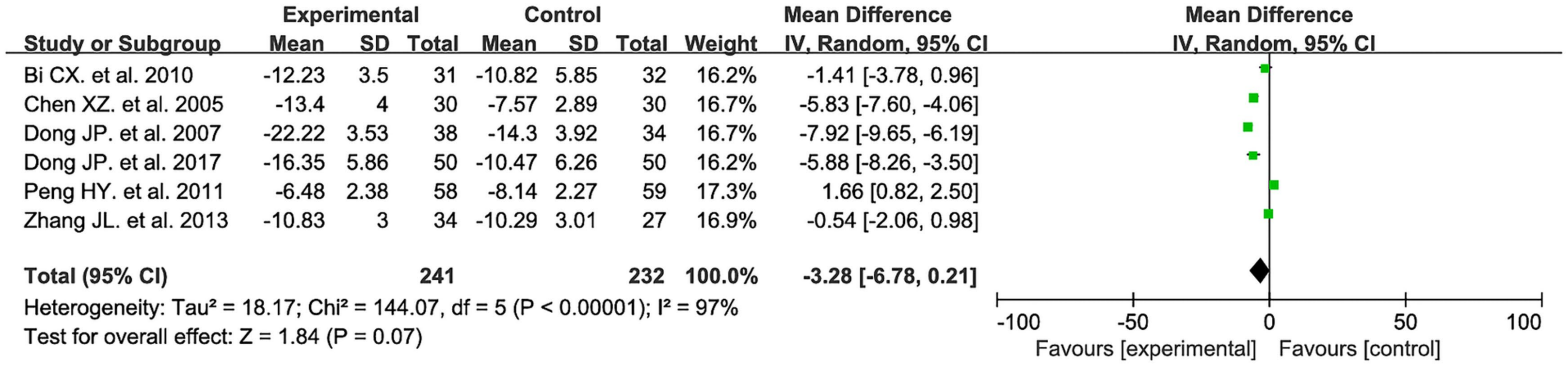
SDS forest plot.

Six studies evaluated SDS before and after treatment, involving 473 patients: 241 in the experimental group and 232 in the control group. Meta-analysis results showed no significant difference between the two groups in improving the Barthel Index (MD = −3.28, 95% CI: −6.78 to 0.21, *I^2^* = 97%, p = 0.07). See Figure 7 for details.

#### 3.5.3 Adverse events

**Figure 8:**
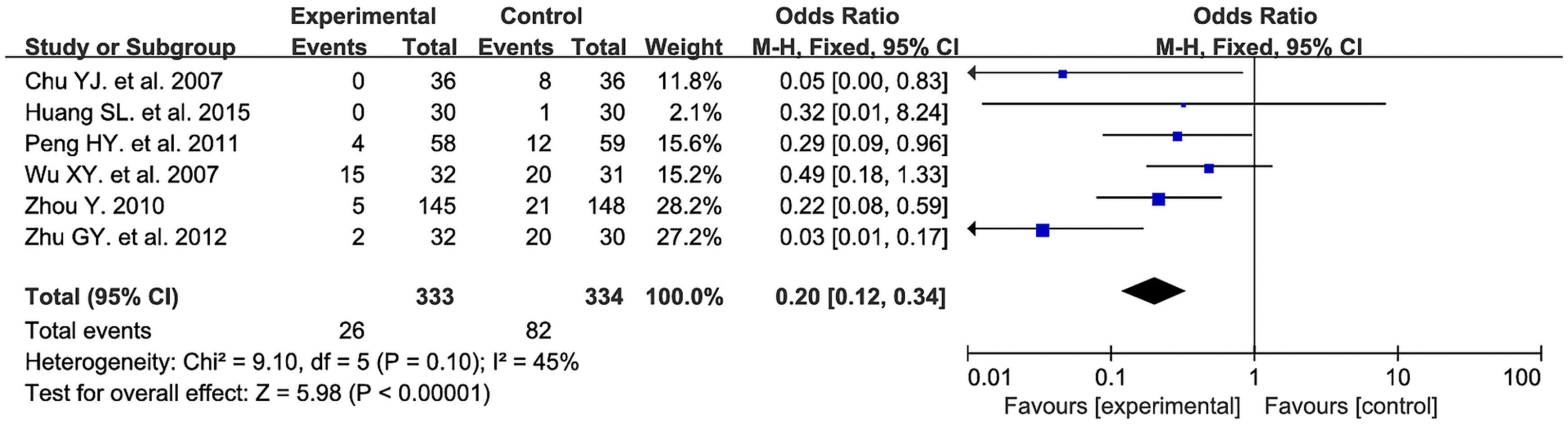
Adverse events forest plot.

Six studies evaluated adverse events before and after treatment, involving 667 patients (333 in the experimental group and 334 in the control group). Meta-analysis results indicated that the experimental group demonstrated superior safety (OR = 0.2, 95% CI: 0.12 to 0.34, *I^2^* = 45%, *p* < 0.00001). See Figure 8 for details.

### 3.6 Bias analysis

**Figure 9:**
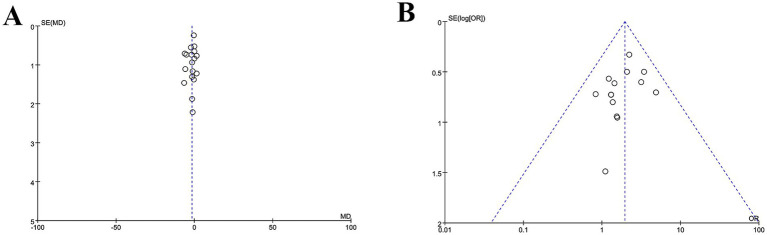
**(A)** HAMD funnel plot. **(B)** Clinical efficacy rate funnel plot.

We examined potential publication bias by plotting funnel plots, where symmetry indicates no publication bias. Figure 9A shows the funnel plot for HAMD, and Figure 9B shows the funnel plot for efficacy rate. As shown in Figure 9A, most studies cluster around effect sizes close to zero, suggesting consistent overall effect directions (no studies with markedly extreme effects). Observing the symmetry of the “funnel” reveals a degree of asymmetry in the distribution of study points (particularly in the region with larger standard errors, where points are skewed toward one side). This asymmetry suggests potential publication bias. As shown in Figure 9B, study points are primarily concentrated in the region where effect sizes are close to 1, indicating that the effect sizes of most studies are consistent in direction. Observing the symmetry of the “funnel” reveals a pronounced asymmetry in the distribution of research points—in regions with larger standard errors (i.e., small-sample studies), the points deviate from the symmetrical funnel shape, exhibiting a distinct “gap” at the bottom. This asymmetry suggests a higher likelihood of publication bias.

**Figure 10:**
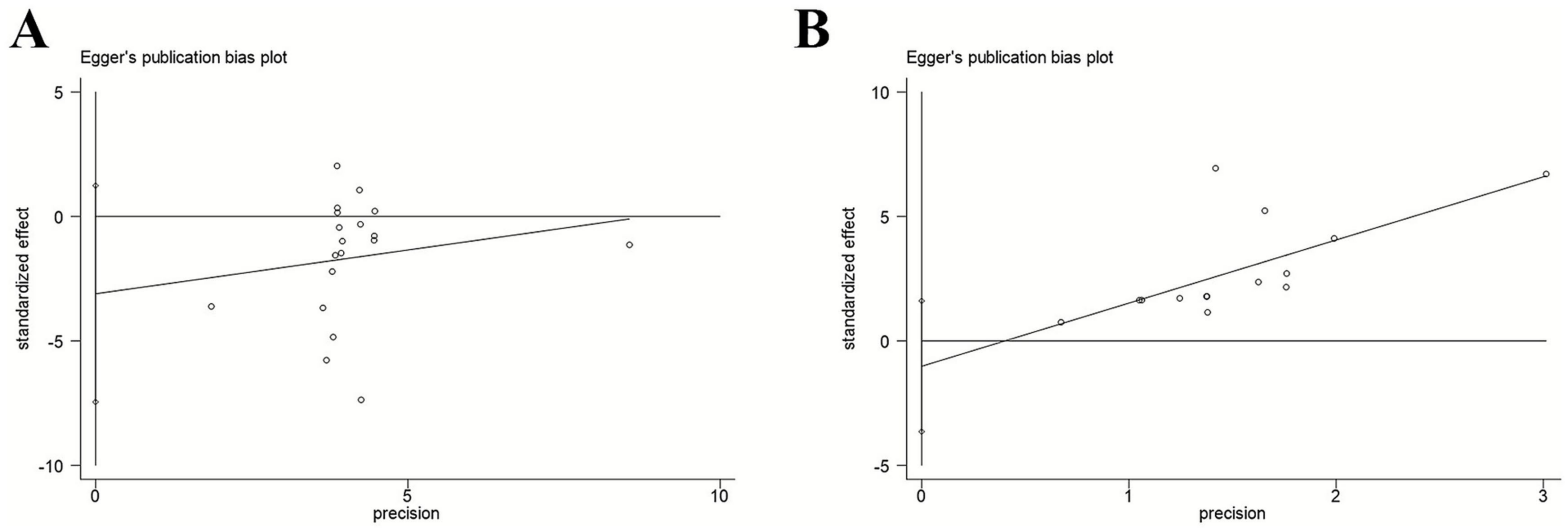
**(A)** The Egger test for HAMD. **(B)** The Egger test for efficacy rate.

To accurately analyse publication bias, we employed the Egger test in Stata 18 to assess the combined effect size of the HAMD. The results indicate that, the Egger test *p*-value for HAMD scores was 0.15, and that for efficacy rate was 0.417, both exceeding the significance level of *α* = 0.05. This indicates no significant linear association between effect size and precision for either measure across the included studies. Furthermore, the scatter plot for the Egger test showed symmetrical distribution of individual studies around the regression line, with no evident skewing. These findings indicate that the included studies in this meta-analysis exhibit no statistically significant publication bias, with a low risk of publication bias. The pooled results for the improvement in HAMD scores and the difference in treatment response rates demonstrate good authenticity and reliability. This provides objective and robust evidence-based medical support for evaluating the clinical efficacy of electroacupuncture in treating post-stroke depression. See Figure 10 for details.

### 3.7 Sensitivity analysis

**Figure 11:**
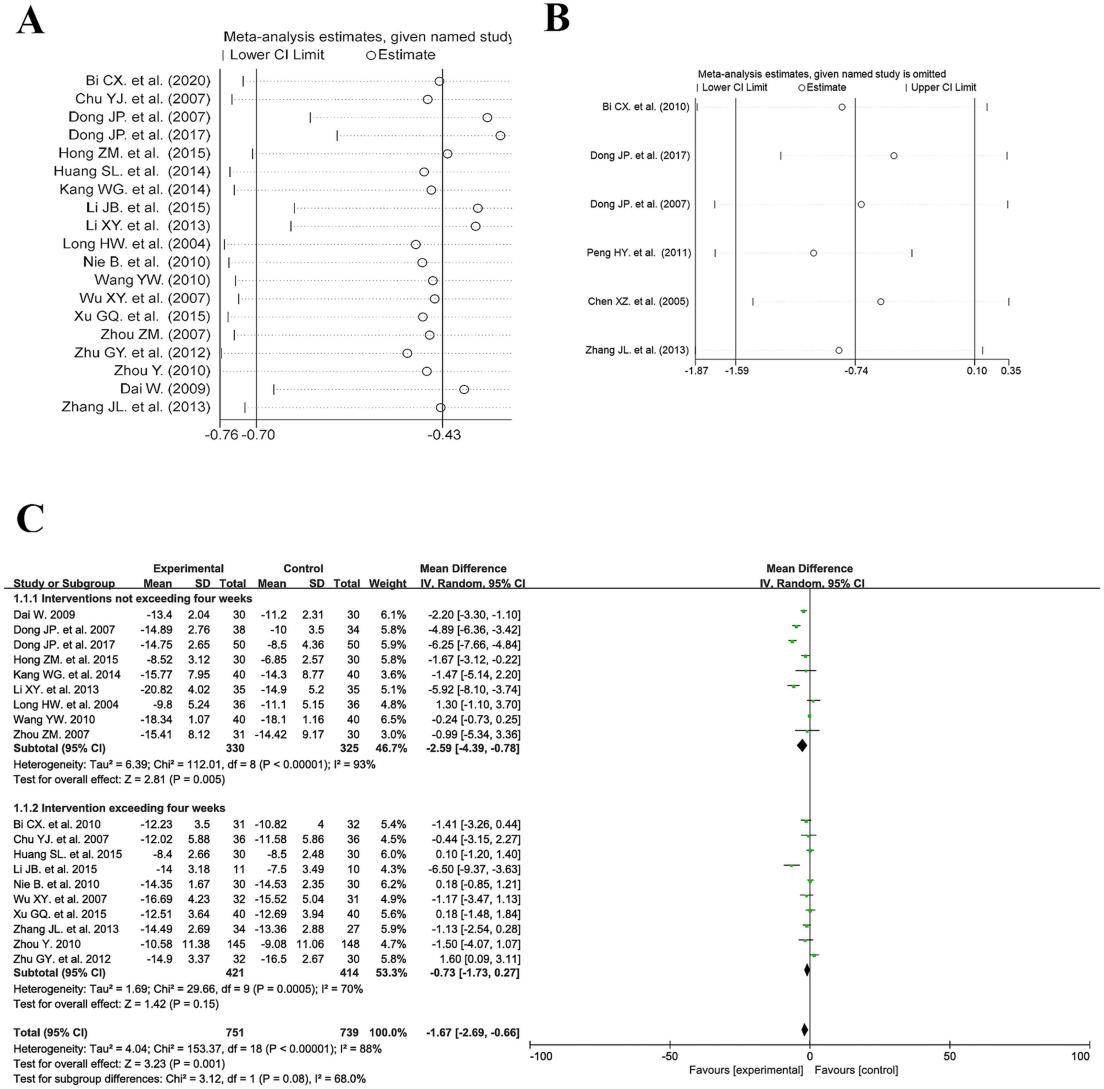
**(A)** HAMD sensitivity analysis. **(B)** SDS sensitivity analysis. **(C)** HAMD subgroup analysis forest plot.

Observation of the meta-analysis results revealed considerable heterogeneity in the HAMD and SDS scores. We conducted sensitivity analyses on both datasets using Stata 18. The findings indicated varying degrees of stability in the outcomes across different endpoints within this study. The analysis using SDS as the outcome measure included six studies. Upon sequentially excluding individual studies, the pooled effect size estimates fluctuated between −1.87 and 0.35. In some instances, the effect size direction reversed and the confidence interval crossed the null line, indicating weak stability in the meta-analysis results for this measure and conclusions susceptible to interference from individual included studies. In contrast, the analysis using HAMD as the outcome measure included 18 studies. After excluding any single study, the pooled effect size consistently remained within the negative range of −0.76 to −0.43. Furthermore, all confidence intervals remained within the non-negligible threshold, with no reversal in effect size direction. This indicates robust stability and high reliability in the meta-analysis results for this measure. This further substantiates the robustness of the conclusion that electroacupuncture intervention demonstrates superior efficacy to conventional pharmacological interventions in ameliorating depressive symptoms (as measured by HAMD scores) in post-stroke patients, with minimal susceptibility to individual study data biases. See Figures 11A,B for details.

To further investigate the sources of heterogeneity, we conducted a subgroup analysis of HAMD-related studies according to treatment duration. The subgroup analysis revealed substantial heterogeneity in both studies with treatment durations not exceeding 4 weeks and those exceeding 4 weeks, suggesting that the sources of heterogeneity may be independent of treatment duration. See Figure 11C for details.

A review of relevant studies revealed inconsistencies between the MDs reported by Huang Shile, Long Haowen, Nie Bin, Xu Guoqing, and Zhu Genying and those from other studies (19, 23, 24, 28, 32). In Huang Shile’s study, the disease duration was significantly shorter than in other studies. In Long Haowen’s study, the control group received an uncertain dosage of 10–40 mg/day, which may have contributed to the heterogeneity. Regarding studies involving the Barthel Index, excluding Hong Zhenmei’s study yielded *I*^2^ = 0%, *p* = 0.1. Reviewing this study revealed that the control group received sertraline tablets, whereas the other two trials used fluoxetine in their control groups, which may explain the heterogeneity.

### 3.8 Evidence grade assessment

We used the GRADE approach to assess the quality of evidence for this study. The meta-analysis results for HAMD and Barthel Index showed very low and low certainty, while meta-analyses for other indicators demonstrated high certainty. Detailed information is provided in Table 3.

**Table 3:** Grade assessment results.

Certainty assessment							No. of patients		Effect		Certainty	Importance
No. of studies	Study design	Risk of bias	Inconsistency	Indirectness	Imprecision	Other considerations	Electroacupuncture	Conventional drugs	Relative (95% CI)	Absolute (95% CI)		
HADM												
18	Randomised trials	Very serious^a^	Not serious^b^	Not serious	Not serious	Publication bias strongly suspected	717	712	—	MD 1.71 lower (2.79 lower to 0.63 lower)	⨁◯◯◯ Very low^a^^,^^b^	
Efficiency												
15	Randomised trials	Not serious^a^	Not serious	Not serious	Not serious	None	616/1227 (50.2%)	611/1227 (49.8%)	OR 1.94 (1.43 to 2.64)	160 more per 1,000 (from 89 more to 226 more)	⨁⨁⨁⨁ High^a^	
Adverse events												
6	Randomised trials	Not serious	Not serious	Not serious	Not serious	None	26/333 (7.8%)	82/334 (24.6%)	OR 0.20 (0.12 to 0.34)	184 fewer per 1,000 (from 208 fewer to 146 fewer)	⨁⨁⨁⨁ High	
SDS												
5	Randomised trials	Not serious	Not serious	Not serious	Not serious	None	241	232	—	MD 3.28 lower (6.78 lower to 0.21 higher)	⨁⨁⨁⨁ High	
Barthel												
3	Randomised trials	Serious^a^	Not serious	Not serious	Not serious	Publication bias strongly suspected	96	96	—	MD 4.01 higher (0.26 lower to 8.27 higher)	⨁⨁◯◯ Low^a^	

CI, confidence interval; MD, mean difference; OR, odds ratio.

a The funnel plot indicates the presence of bias.

b The meta-analysis results exhibit heterogeneity.

## 4 Discussion

This systematic review and meta-analysis evaluated the efficacy and safety of EA for treating PSD. Results indicate that EA outperformed conventional drug therapy in improving HAMD scores, increasing treatment response rates, and reducing adverse events. However, no significant differences were observed between EA and drug groups in improving Barthel Index or SDS scores. These findings provide preliminary evidence-based support for incorporating EA into the clinical management of PSD.

### 4.1 Potential mechanisms of EA in treating PSD

#### 4.1.1 Regulation of mitochondrial function

In recent years, multiple studies have confirmed the efficacy of EA for PSD and identified potential mechanisms. Researchers found that EA increases AMPK expression in the prefrontal cortex of PSD rats, thereby improving mitochondrial function and alleviating depressive symptoms in model rats (35). In another study, researchers confirmed that EA intervention promotes cannabinoid receptor 1 (CB1R) and mitochondrial expression, restores mitochondrial dysfunction, and consequently improves PSD (36).

#### 4.1.2 Regulation of monoamine neurotransmitters and neurotrophic factors

Multiple studies suggest that EA may exert antidepressant effects by modulating serotonin system function. Xu Nenggui and Yao Lulu’*s team* published findings in *Translational* Psychiatry indicating that electrical EA stimulation at Baihui and Shenting acupoints significantly enhances 5-HT neural projections from the dorsal raphe nucleus to the prefrontal cortex, restoring functional connectivity in this pathway and thereby improving depressive-like behaviors in PSD mice. Further chemogenetic inhibition experiments demonstrated that the DRN-mPFC 5-HT neural circuit is essential for EA efficacy. When this circuit was specifically suppressed, EA’s therapeutic effects were markedly attenuated (37). This finding strongly aligns with the significant improvement in HAMD scores observed in the EA group within this meta-analysis, suggesting a distinct neural circuit regulatory mechanism underlies clinical efficacy.

EA may also exert effects by modulating brain-derived neurotrophic factor (BDNF). Researchers have found that EA applied to the Four Gates points promotes hippocampal BDNF and TrkB expression in PSD rats (38). Further studies revealed that EA enhances serum and prefrontal cortex BDNF expression in model rats. Additionally, EA was shown to increase tPA and TrkB expression in the prefrontal cortex of PSD rats, proposing that EA improves PSD by regulating the tPA/BDNF/TrkB pathway (39).

#### 4.1.3 Regulation of inflammation and ferroptosis

Inflammation is closely associated with PSD. Studies have revealed that EA exerts its effects by regulating inflammation and ferroptosis. Li′s research demonstrated that EA reduces inflammatory factor expression in model mice, protects hippocampal neurons, and lowers TLR4, p-p38, p-NF-κB, and NLRP3 levels, validating the mechanism whereby EA acts through the TLR4/p38/NF-κB/NLRP3 pathway (40). Recent studies further confirm that EA alleviates hippocampal inflammation and pyroptosis in PSD by downregulating NLRP3 inflammasome activation, suggesting NLRP3 as a potential therapeutic target for PSD (41). The association between ferroptosis and depression has been established (42), and the mechanism by which EA improves PSD through ferroptosis has been elucidated. Gao’s study revealed that EA inhibits ferroptosis in prefrontal cortical neurons to reduce neurological deficits and enhance spontaneous activity and exploratory behavior in rats (43).

In summary, current research has confirmed the definite efficacy of EA in treating PSD and identified multiple targets and pathways through which EA exerts its effects, demonstrating systemic and holistic characteristics.

### 4.2 Overview of meta-analysis results

Notably, the pooled results for HAMD scores and SDS in this study exhibited high heterogeneity (*I*^2^ = 89%; *I*^2^ = 97%), indicating significant clinical or methodological differences among studies. Sensitivity analysis revealed that inconsistencies in patient disease duration, drug dosage, and types were likely primary sources of heterogeneity. For example, the shorter disease duration in Huang et al.’s (19) study and the wider range of drug dosages in the control group of Long et al.’s (23) study may have impacted the consistency of results. Future studies should standardize intervention protocols and patient stratification to enhance the reliability of findings.

Regarding secondary outcomes, no significant differences were observed between EA and medication in improving the Barthel Index and SDS scores. This may stem from limited sample sizes or varying sensitivities of assessment tools across different dimensions of psychological and functional status. Furthermore, the Barthel Index primarily reflects activities of daily living, whereas the SDS is a self-report scale; its assessment of depression severity may differ from clinician-rated HAMD scores.

Furthermore, all included studies were conducted in China, and most exhibited suboptimal methodological quality, particularly with inadequate implementation of blinding and allocation concealment, potentially introducing implementation and measurement biases. The findings of this study are primarily applicable to the Chinese population and should be applied with caution to other regions or populations. Future research requires more high-quality, multicenter, large-sample randomized controlled trials to enhance the generalizability and strength of evidence.

Furthermore, this study applied more precise inclusion criteria for the experimental group. For the experimental group, the studies included in our analysis featured subjects receiving only EA treatment, while the control groups received only conventional drug therapy. This allows for a more precise analysis of the advantages and limitations of acupuncture compared to conventional therapies. Previous meta-analyses (44), however, appear to have applied broader criteria for intervention methods, incorporating studies using various acupuncture techniques such as EA, body acupuncture, auricular acupuncture, and scalp acupuncture. While this approach yielded more comprehensive results, it lacked the necessary precision.

## 5 Limitations

This study has several limitations. First, all included studies originated from China, potentially introducing regional bias and limiting the generalizability of findings. Second, most studies inadequately described specific methods for random sequence generation and allocation concealment, and incomplete blinding may have introduced implementation and measurement biases. Furthermore, the number of studies for certain outcome measures was limited—for example, only three studies included the Barthel Index—compromising the robustness of results. Finally, funnel plots suggest potential publication bias, particularly with small-sample studies showing a tendency toward positive results, indicating that negative findings may be underreported.

## 6 Conclusion

This systematic review of existing clinical trials preliminarily confirms the efficacy of EA in improving core PSD symptoms and its safety profile. Future research should focus on multicenter, large-sample, methodologically rigorous randomized controlled trials to further validate the long-term efficacy and mechanisms of EA, and to explore response variations across different patient subgroups.

## Data Availability

The original contributions presented in the study are included in the article/Supplementary material, further inquiries can be directed to the corresponding author.
