# Supplementary material for: Efficacy and safety of electroacupuncture for post-stroke depression: a systematic review and meta-analysis
Source: Front Neurol. 2026 Jan 12;16:1732787. doi: 10.3389/fneur.2025.1732787 (PMC12832317; doi:10.3389/fneur.2025.1732787)
Supplement: Supplementary file 1 [file Table_1.DOCX]

Table 1 Search Strategy of Web of Science

| Rank | Search term | Result |
| --- | --- | --- |
| #1 | Electroacupuncture OR Electroacupuncture OR electro-acupuncture OR electric acupuncture OR Electroneedle OR “Electroacupuncture Treatment” OR “Treatment, Electroacupuncture” | 14026 |
| #2 | Stroke OR Ischemic Stroke OR Hemorrhagic Stroke OR Brain Infarction OR Cerebral Hemorrhage OR Cerebrovascular Accident OR Cerebrovascular Accidents OR CVA OR Cerebrovascular Apoplexy OR Apoplexy, Cerebrovascular OR Vascular Accident, Brain OR Brain Vascular Accident* OR Vascular Accidents, Brain OR Cerebrovascular Stroke* OR Stroke, Cerebrovascular OR Strokes, Cerebrovascular OR Apoplexy OR Cerebral Stroke* OR Stroke, Cerebral OR Strokes, Cerebral OR Stroke, Acute OR Acute Stroke* OR Strokes, Acute OR Cerebrovascular Accident, Acute OR Acute Cerebrovascular Accident* OR Cerebrovascular Accidents, Acute | 784243 |
| #3 | Depression OR Depressive Disorder OR Depressive Symptoms OR Depressive Symptom OR Symptom, Depressive OR Emotional Depression OR Depression, Emotional OR Depressive Disorders OR Disorder*, Depressive OR Neurosis, Depressive OR Depressive Neuroses OR Depressive Neurosis OR Neuroses, Depressive OR Depression*, Endogenous OR Endogenous Depression* OR Depressive Syndrome* OR Syndrome*, Depressive OR Depression*, Neurotic OR Neurotic Depression* OR Melancholia* OR Unipolar Depression OR Depression*, Unipolar OR Unipolar Depressions | 1023188 |
| #4 | #1 AND #2 AND #3 | 68 |

Table 2 Search Strategy of Embase

| Rank | Search term | Result |
| --- | --- | --- |
| #1 | Electroacupuncture OR Electroacupuncture OR electro-acupuncture OR electric acupuncture OR Electroneedle OR “Electroacupuncture Treatment” OR “Treatment, Electroacupuncture” | 13296 |
| #2 | Stroke OR Ischemic Stroke OR Hemorrhagic Stroke OR Brain Infarction OR Cerebral Hemorrhage OR Cerebrovascular Accident OR Cerebrovascular Accidents OR CVA OR Cerebrovascular Apoplexy OR Apoplexy, Cerebrovascular OR Vascular Accident, Brain OR Brain Vascular Accident* OR Vascular Accidents, Brain OR Cerebrovascular Stroke* OR Stroke, Cerebrovascular OR Strokes, Cerebrovascular OR Apoplexy OR Cerebral Stroke* OR Stroke, Cerebral OR Strokes, Cerebral OR Stroke, Acute OR Acute Stroke* OR Strokes, Acute OR Cerebrovascular Accident, Acute OR Acute Cerebrovascular Accident* OR Cerebrovascular Accidents, Acute | 1699 |
| #3 | Depression OR Depressive Disorder OR Depressive Symptoms OR Depressive Symptom OR Symptom, Depressive OR Emotional Depression OR Depression, Emotional OR Depressive Disorders OR Disorder*, Depressive OR Neurosis, Depressive OR Depressive Neuroses OR Depressive Neurosis OR Neuroses, Depressive OR Depression*, Endogenous OR Endogenous Depression* OR Depressive Syndrome* OR Syndrome*, Depressive OR Depression*, Neurotic OR Neurotic Depression* OR Melancholia* OR Unipolar Depression OR Depression*, Unipolar OR Unipolar Depressions | 1560 |
| #4 | #1 AND #2 AND #3 | 74 |

Table 3 Search Strategy of Cochrane Library

| Rank | Search term | Result |
| --- | --- | --- |
| #1 | Electroacupuncture OR Electroacupuncture OR electro-acupuncture OR electric acupuncture OR Electroneedle OR “Electroacupuncture Treatment” OR “Treatment, Electroacupuncture” | 5436 |
| #2 | Stroke OR Ischemic Stroke OR Hemorrhagic Stroke OR Brain Infarction OR Cerebral Hemorrhage OR Cerebrovascular Accident OR Cerebrovascular Accidents OR CVA OR Cerebrovascular Apoplexy OR Apoplexy, Cerebrovascular OR Vascular Accident, Brain OR Brain Vascular Accident* OR Vascular Accidents, Brain OR Cerebrovascular Stroke* OR Stroke, Cerebrovascular OR Strokes, Cerebrovascular OR Apoplexy OR Cerebral Stroke* OR Stroke, Cerebral OR Strokes, Cerebral OR Stroke, Acute OR Acute Stroke* OR Strokes, Acute OR Cerebrovascular Accident, Acute OR Acute Cerebrovascular Accident* OR Cerebrovascular Accidents, Acute | 87298 |
| #3 | Depression OR Depressive Disorder OR Depressive Symptoms OR Depressive Symptom OR Symptom, Depressive OR Emotional Depression OR Depression, Emotional OR Depressive Disorders OR Disorder*, Depressive OR Neurosis, Depressive OR Depressive Neuroses OR Depressive Neurosis OR Neuroses, Depressive OR Depression*, Endogenous OR Endogenous Depression* OR Depressive Syndrome* OR Syndrome*, Depressive OR Depression*, Neurotic OR Neurotic Depression* OR Melancholia* OR Unipolar Depression OR Depression*, Unipolar OR Unipolar Depressions | 126469 |
| #4 | #1 AND #2 AND #3 | 79 |
